# Identification of new members of hydrophobin family using primary structure analysis

**DOI:** 10.1186/1471-2105-7-S4-S16

**Published:** 2006-12-12

**Authors:** Kuan Yang, Youping Deng, Chaoyang Zhang, Mohamed Elasri

**Affiliations:** 1 Department of Biological Sciences, University of SouthernMississippi, Hattiesburg, Mississippi 39406, USA; 2 School of Computing, University of Southern Mississippi, Hattiesburg, Mississippi 39406, USA

## Abstract

**Background:**

Hydrophobins are fungal proteins that can turn into amphipathic membranes at hydrophilic/hydrophobic interfaces by self-assembly. The assemblages by Class I hydrophobins are extremely stable and possess the remarkable ability to change the polarity of the surface. One of its most important industrial applications is its usage as paint. Without detailed knowledge of the 3D structure and self-assembly principles of hydrophobins, it is difficult to make significant progress in furthering its research.

**Results:**

In order to provide useful information to hydrophobin researchers, we analyzed primary structure of hydrophobins to gain more insight about these proteins. In this paper, we presented an in-depth primary sequence analysis using batch BLAST search of the database, sequence filtering by programming and motif finding by MEME. We used batch BLAST to find similar sequences in the NCBI nr database. Then we used MEME to find out motifs. Based on the newly found motifs and the well-known C-CC-C-C-CC-C pattern we used MAST to search the entire nr database. At the end, domain search and phylogenetic analysis were conducted to confirm the result. After searching the nr database with the new PSSM-format motifs identified by MEME, many sequences from various species were found by MAST. Filtering process by pattern, domain and length left 9 qualified candidates.

**Conclusion:**

All of 9 newly identified potential hydrophobins possess the common pattern and hydrophobin domain. From the multiple sequence alignment result, we can see that some of them are grouped very close to other known hydrophobins, which means their phylogenetic relationship is very close and it is highly plausible that they are indeed hydrophobin proteins.

## Background

Hydrophobins play a very important role in fungal growth and development. They are a class of small fungal proteins, which are responsible for the formation of aerial hyphae and attachment of hyphae to hydrophobic surfaces [1]. The remarkable ability of self-assembly at hydrophilic/hydrophobic surfaces makes hydrophobins interesting and unique, not only in biology but also in industrial applications. There are two main classes of hydrophobins: class I and class II. These two classes have been distinguished based on the differences in their hydropathy patterns and biophysical properties [2]. The membrane formed by self-assembly of Class I hydrophobins is more stable than the one formed by Class II. It is also characterized by a rodlet structure, which does not exist in Class II hydrophobins. More interestingly, this structure is observed in amyloid proteins, which suggests similarities in structure and self-assembly mechanism.

SC3, a glycosylated hydrophobin, is the most extensively studied class I hydrophobin to date [3]. It contains the only known motif in hydrophobins, the eight-cysteine residues [4]. The cysteines form four disulfide bridges, which keep SC3 from self-assembling and account for controlled assembly at hydrophilic/hydrophobic interfaces [5]. They also divide the entire molecule into four loops with N-terminal sequence of nearly 30 amino acids. On the other end of the sequence, the C-terminal region only contains 6 amino acids [6].

It is assumed that self-assembly of class I hydrophobins is accompanied by conformational changes as it is the case for SC3. The intermediate α-helix state changes into the stable ultimate β-sheet form at water-air interface. However, study of class I hydrophobin EAS from Neurospora crassa by NMR found no obvious structure other than a small core region composed of three antiparallel β-strands, which is probably stabilized by the four disulfide bridges [7]. This discrepancy in both structure and sequence is a major hurdle in studying hydrophobins and their unique self-assembly properties. In this paper, we use computational tools to extract information from hydrophobin sequences and identify conserved motifs to find new members of the hydrophobin family.

## Results

### Batch BLAST search for all hydrophobin sequences and identification of new conserved motifs

A total of 183 sequences containing the key word hydrophobin were found including 22 sequences in UniProtKB/Swiss-Prot and 161 sequences in UniProtKB/TrEMBL, most of which were fragments only. No filtering was performed only because we did not want to lose any potential signals. All 183 sequences, including fragments, were used to conduct batch BLAST search against nr database on NCBI server. A perl program was used to perform this task. By doing so, all potential hydrophobins should have been found in one or more search results. 6715 sequences were returned by the batch BLAST. All results were automatically filtered by the presence of the word hydrophobin in their description. After elimination of identical sequences, judged by GI number, and the sequences that don't have the eight-cysteine residues pattern (C-CC-C-C-CC-C) by the perl program, 128 hydrophobin non-fragment sequences were dug out from the nr database and put into an Excel file. Also, the program retrieved all relative information on each individual sequence of these 128 hydrophobins to prepare for the conserved motif search.

Highly similar sequences were removed to reduce bias on conserved motif search. Only 110 sequences were used as input to MEME to conduct conserved motif search. Considering the high diversity among the hydrophobin sequences, the maximum motif number was set to 10. The first 6 motifs were reasonable and therefore used as input to MAST program. They are in PSSM format; however the consensus sequences are shown in the Table 1.

### Identification of new hydrophobins

Using motifs obtained from MEME, we conducted a MAST search and analyzed the results with a perl program. It removed not only all the identical sequences but also the ones used as input from the search results. The e-value for qualified sequence is 10.

As we have mentioned before, in order not to miss any signal, we have set the maximum motif number to 10, which leads to the increase in the number of false positives. Pattern matching and domain analysis have been conducted to filter the results from the last step. First, any known hydrophobin sequences in the returned sequences were removed (111 sequences removed). Then, all the obtained sequences have been scanned with the eight-cysteine-residue pattern (19 sequences removed). All sequences containing more or less cysteine residues have been filtered out. The sequence gi:71020699 hypothetical protein was also filtered out because its length is far larger than any other known hydrophobin sequence. At last, domain analysis with SMART on each individual sequences was conducted. The existence of hydrophobin domain has to be positive to keep the sequence in the candidate list (6 sequences removed).

In total, 9 strong candidates as potential hydrophobins were found among five species, Aspergillus nidulans, Agrocybe aegerita, Gibberella zeae, Metarhizim anisopliae and Ustilago maydis. The details are listed in Table 2. These nine hydrophobins have not been named as hydrophobins and not reported previously, and are found for the first time in the study.

### Pattern and domain analysis

**Figure 1:**
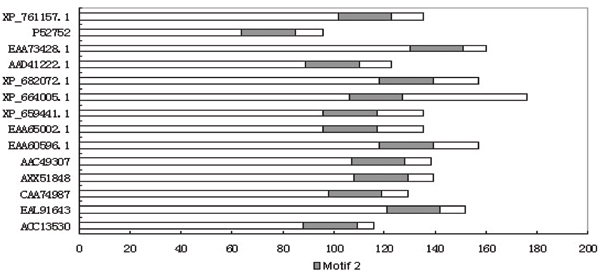
**Motif Alignment among known and newly identified hydrophobins**. The second motif we found exists in almost all the hydrophobin sequences. The first 9 sequences are the new identified hydrophobins and the other 5 sequences are known hydrophobins.

**Figure 2:**
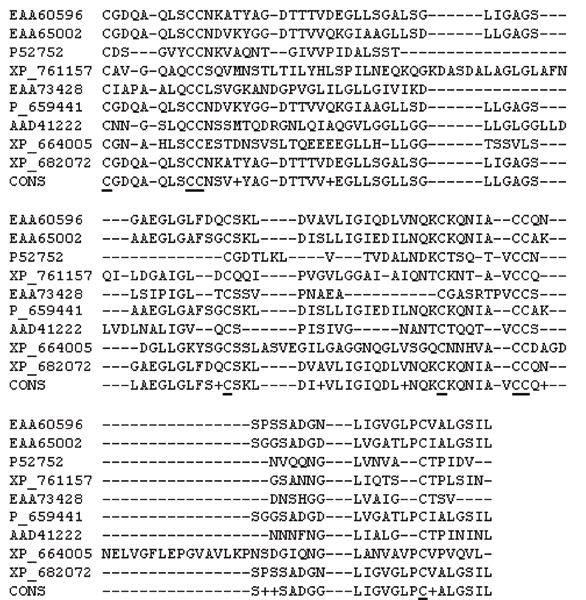
The alignment of the regions including the eight-cysteine-residues in the 9 sequences.

**Figure 3:**
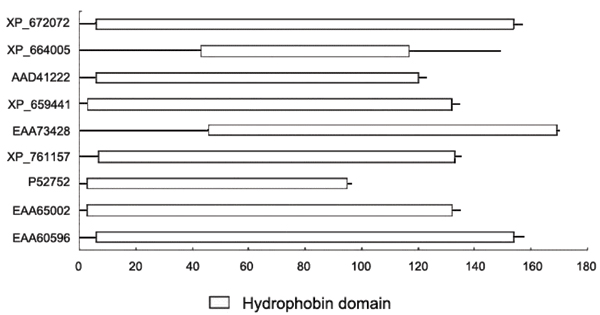
**Hydrophobin Domain in the newly identified sequences**. All the 9 newly identified hydrophobins contain the hydrophobin domain stored in the Pfam database. This is another indication that they are possibly hydrophobins.

As we described, conserved motifs were used to search for potential novel hydrophobins, pattern and domain analysis were further used to filter the results. Consequently, all these 9 new hydrophobin sequences have at least one of the 6 conserved motifs we first found in this study. In Figure 1, we aligned the second motif in these 9 sequences and other known hydrophobin sequences (5 sequences), and this motif is highly conserved among all the sequences including new and known hydrophobin sequences, indicating the motifs we identified in the study are useful for finding potential new hydrophobins. We also did a multiple sequence alignment (figure 2) which clearly shows the hydrophobin pattern in all the newly identified hydrophobin sequences. In figure 3, we can clearly see the detail location of the hydrophobin domain in each newly identified hydrophobin sequence.

### Phylogenetic tree

**Figure 4:**
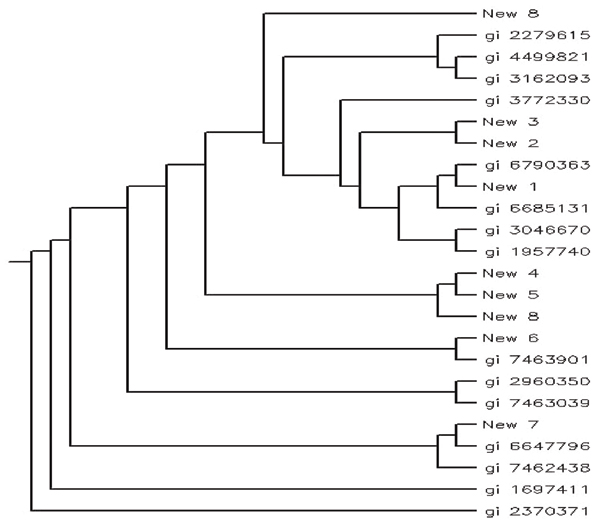
**Phylogenetic analysis among newly identified hydrophobins and other known hydrophobins**. In this figure we can see that newly identified hydrophobins are grouped together with other known hydrophobins, which strongly supports the research.

All the potential hydrophobins found in our analysis were used to build a phylogenetic tree that also included selected known hydrophobins. The purpose of phylogenetic analysis is to study the evolutionary relationship between the hydrophobins identified here and the other known hydrophobins and also to perform a final check on the reliability of the results obtained. We selected 15 sequences from the input sequence pool along with 9 new identified sequences to construct a phylogenetic tree. The results are shown in Figure 4. We can see that most new identified hydrophobins are grouped together with other reference sequences, which supports our results.

## Discussion

The unique property of hydrophobins to self-assemble at hydrophobic-hydrophilic interfaces makes them interesting candidates for use in medical and industrial applications [8]. The high stability of the Class I hydrophobins have made them ideal candidates for industrial application. For example, hydrophobins could be used as a protection layer of many instruments against damage from other chemical reagents. It could also be used to change a hydrophobic surface into a hydrophilic one and vice versa. In addition, the size and properties of rodlets formed by class I hydrophobins make them useful for application in nanotechnology. Another very important application is the use of hydrophobins as an intermediate to attach cells or molecules to surfaces with different properties. Each hydrophobin is slightly different from the others, which makes it possible to find a suitable hydrophobin for a specific application. This underlines the need to identify new hydrophobins.

The best way of making use of hydrophobins is structure determination. In order to facilitate research in hydrophobin structure, a database is currently under development. This database will store not only all common protein properties but also properties specific to hydrophobins, such as self-assembled layer stability. Users can make various queries against the database, such as all the hydrophobin sequences in a specific species. With more and more hydrophobins discovered, more information will be answered by sequence analysis.

## Conclusion

All of 9 newly identified potential hydrophobins possess the common pattern and hydrophobin domain. The result of the multiple sequences alignment has shown that some of the newly identified potential hydrophobin sequences are closely grouped to other known ones, which strongly supported the result of this research. The phylogenetic relationships between them are very close. Besides, we have also identified a pattern which exists in almost all hydrophobin sequences. It could become a very accurate criterion in new hydrophobin identification and it could also facilitate the research on the unique property of hydrophobin family.

## Methods

**Figure 5:**
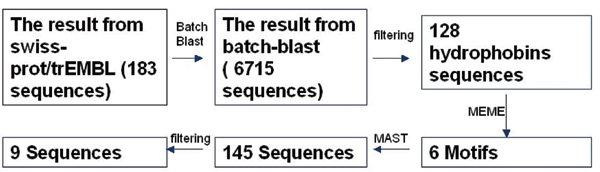
Workflow of the entire project.

The entire workflow is given as following (Figure 5). It gives an overview of the methods used in this paper.

### Prepare sequences

Key word hydrophobin was used to search the UniProt Knowledgebase (Swiss-Prot and TrEMBL ) to find existing hydrophobin protein sequences. Both whole length sequences and fragments are included to insure the maximum coverage of hydrophobin sequence information.

### Batch BLAST

A perl program was used to conduct batch BLAST against NCBI database . In order to keep noise at the lowest level, all qualified hydrophobin sequences contained the eight-cysteine residue pattern (C-CC-C-C-CC-C) along with word hydrophobin in their sequence description. The perl program also conducted filtering based on the criteria.

### Multiple Sequence alignment and highly identical sequences elimination

Multiple sequence alignment was performed using ClustalW program  available at the European Bioinformatics Institute web site [9]. Based on the result, highly identical sequences were removed to reduce the bias on the conserved motif search.

### Conserved motif search

MEME [10] and PRATT [11] were used to dig out conserved motif information. The output of the program is completed studied and then used as input to the next step of the pipeline.

### Database search for new hydrophobins

MAST [12], bundled with MEME, was used to search for new hydrophobin sequences against nr database (non-redundant protein database). Huge amount of sequences are retrieved. A perl program is written to eliminate replications based on genbank ID.

### Domain analysis of identified hydrophobins

SMART [13] domain analysis tool was used not only to predict the domain architecture of identified sequences but also to filter the results. A domain alignment between the newly identified potential hydrophobins and the known hydrophobins is conducted based on the result of this research.

### Phylogenetic analysis

Phylip  was used to build a phylogenetic tree. A phylogenetic tree was constructed using the neighbor-joining algorithm as described by Saitouand Nei [14]. To investigate the evolutionary relationship between the putative hydrophobins identified and other hydrophobins, a phylogenetic tree was built.

## Authors' contributions

KY carried out pattern analysis, batch-blast search, domain identification and analysis, phylogenetic analysis and programming. YD gave general direction and paper revise. ME participated in paper modification.

## Figures and Tables

**Table 1:** List of Consensus Sequences of the 6 motifs.

**Motif No.**	**Consensus sequences**
1	KCGDQAQLSCCNKATYAQDVTDIDEFILAGTLKNLIGGGSG{T, S}EGLGLF{D, N}Q
2	{D, G}L{V, G}{G, N}Q{K, S}C{K, S}{Q, A}{Q, N}{I, T}{V, A}CCQN{S, N}{P{F, S}{D, N}{G, A}
3	{S, Q}{Q, C}{C, S}{N, Q}{T, G}{G, Q}{T, S}{L, V, A}{Q, K}CCNS
4	VQS{A, S}S{S, D, N}PX{V, A}{A, G}{G, L}LLGLLG{I, V}V{L, V}G
5	L{V, I}{G, N}LTC{S, T}PI{S, T}V
6	SX{T, V}A{A, L}VLALAA{A, L}{A, L}{A, V}{A, V}AXPXPX

6 motifs are selected to search the NCBI nr database. All of these 6 motifs exist in at least 1 of the returned sequences. This raised the possibility of retrieving the positive signal as well as the noise. Pattern and domain analysis were conducted to compensate this side effect, which will be described later.

**Table 2:** All the identified new hydrophobins and their source organisms.

**GenBank Accession No.**	**Locus_tag/Gene Name**	**Length**	**Source Organism**	**GI**
EAA60596.1	AN8803.2	157	Aspergillus nidulans	40741406
EAA65002.1	AN1837.2	135	Aspergillus nidulans	40745846
XP_659441.1	AN1837.2	135	Aspergillus nidulans	67522761
XP_664005.1	AN6401.2	162	Aspergillus nidulans	67540462
XP_682072.1	PB401492	157	Aspergillus nidulans	67903632
AAD41222.1	pri2	123	Agrocybe aegerita	5256969
EAA73428.1	FG03960.1	170	Gibberella zeae	42550585
P52752	SSGA	96	Metarhizium anisopliae	1711536
P_761157.1	UM05010.1	135	Ustilago maydis	71021853

This raised the possibility of retrieving the positive signal as well as the noise. Pattern and domain analysis were conducted to compensate this side effect, which will be described later.
